# (1*S*,2*S*,5*S*)-2-Methyl-3-oxo-5-(prop-1-en-2-yl)cyclo­hexane-1-carbo­nitrile

**DOI:** 10.1107/S1600536813011197

**Published:** 2013-04-27

**Authors:** Marcos L. Rivadulla, Alioune Fall, María González, Maria J. Matos

**Affiliations:** a Departamento de Química Orgánica, Facultade de Química, Universidade de Vigo, E-36310 Vigo, Spain; b Departamento de Química Orgánica, Facultade de Farmacia, Universidade de Santiago de Compostela, 15782 Santiago de Compostela, Spain

## Abstract

The mol­ecule of the title compound, C_11_H_15_NO, contains a cyclo­hexa­none ring, three defined stereocenters and an exocyclic double bond. The crystal structure is the result of a study on the Michael addition reaction of (*S*)-carvone with sodium cyanide using ionic liquids as the reaction medium and so the absolute configuration is known from the chemistry. The six-membered ring is in a chair conformation.

## Related literature

For recent review of Ionic liquids as solvents, see: Welton (1999 ▶); Wasserscheid & Keim (2000 ▶).
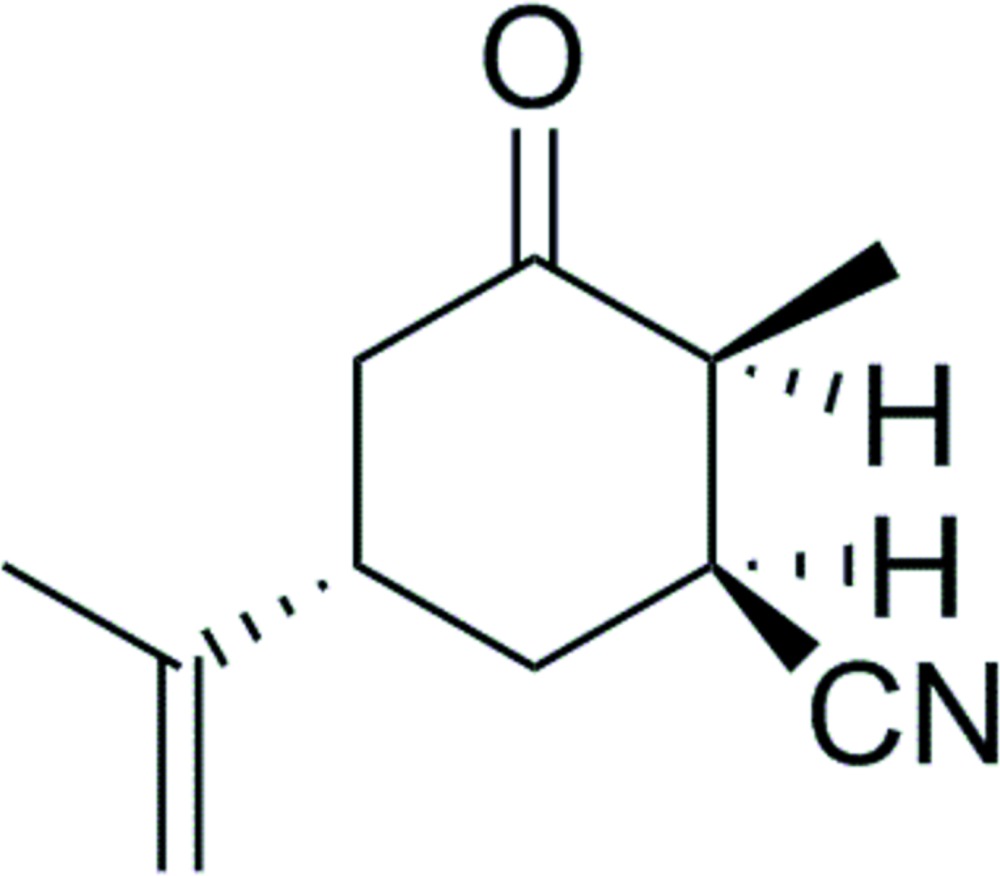


## Experimental

### 

#### Crystal data

C_11_H_15_NO
*M* *_r_* = 177.24
Orthorhombic, 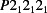

*a* = 5.2892 (8) Å
*b* = 10.7213 (16) Å
*c* = 19.559 (3) Å
*V* = 1109.1 (3) Å^3^
*Z* = 4
Mo *K*α radiation
μ = 0.07 mm^−1^
*T* = 293 K
0.60 × 0.56 × 0.42 mm

#### Data collection

Bruker SMART 1000 CCD diffractometer
Absorption correction: multi-scan (*SADABS*; Sheldrick, 1995 ▶) *T*_min_ = 0.861, *T*_max_ = 1.000
7138 measured reflections
2590 independent reflections
2112 reflections with *I* > 2σ(*I*)
*R*_int_ = 0.023

#### Refinement

*R*[*F* ^2^ > 2σ(*F* ^2^)] = 0.052
*wR*(*F* ^2^) = 0.164
*S* = 1.04
2590 reflections
120 parameters
H-atom parameters constrained
Δρ_max_ = 0.24 e Å^−3^
Δρ_min_ = −0.20 e Å^−3^

### 

Data collection: *SMART* (Bruker, 2007 ▶); cell refinement: *SAINT* (Bruker, 2007 ▶); data reduction: *SAINT*; program(s) used to solve structure: *SHELXS97* (Sheldrick, 2008 ▶); program(s) used to refine structure: *SHELXL97* (Sheldrick, 2008 ▶); molecular graphics: *SHELXTL* (Bruker, 2007 ▶); software used to prepare material for publication: *PLATON* (Spek, 2009 ▶).

## Supplementary Material

Crystal structure: contains datablock(s) I, global. DOI: 10.1107/S1600536813011197/go2087sup1.cif

Structure factors: contains datablock(s) I. DOI: 10.1107/S1600536813011197/go2087Isup2.hkl

Supplementary material file. DOI: 10.1107/S1600536813011197/go2087Isup3.cml

Additional supplementary materials:  crystallographic information; 3D view; checkCIF report

## Figures and Tables

**Fig. 1.**
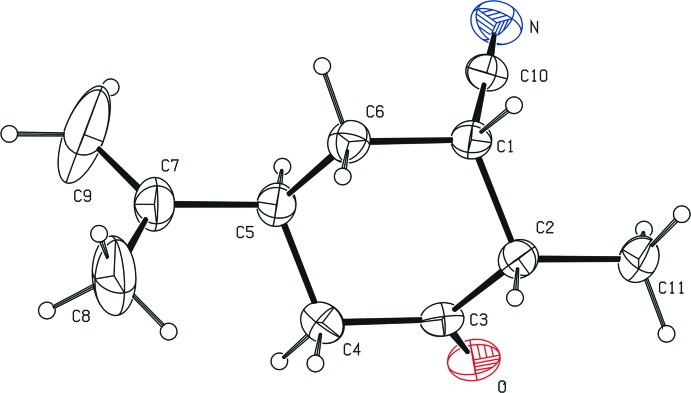
The molecular structure of the title compound with the atom-numbering scheme. Displacement ellipsoids are drawn at the 30% probability level.

## supplementary crystallographic information

### Comment

Ionic Liquids (ILs) have been attracting considerable attention in the last decade as a new media due to their unique physical and chemical properties (Welton, 1999, Wasserscheid and Keim, 2000). They are often preferred as being more environmentally friendly than traditional organic solvents. The range of known and available ILs has been rapidly growing and nowadays many ILs are commercially available. In accordance with current trends in academic and industrial research, in recent years our research group has also began to work towards the replacement of toxic volatile organic solvents with ILs. In the title compound, I, (Fig. 1), it is observed that the six-membered ring adopts the usual chair conformation. The C1–C2 bond of the cyclohexanone moiety adopts a *cis* configuration. The dihedral angle between the main plane of the cyclohexanone ring (defined for C1–C3–C4–C6) and the main plane of the lateral chain (defined for C5–C7–C8–C9) is 73.97°. The C5–C7–C8–C9 atoms of the lateral chain are co-planar but show large thermal motion. The absolute configuration was established according to the configuration of the starting material.

### Experimental

Over a stirring solution of (S)-(+)-Carvone (104µ*L*; 0.67 mmol) in the ionic liquid [TMG][LAC] (1 mL), NaCN (39.15 mg; 0.8 mmol) was added. The mixture was stirred at 60 °C for 12 h. Then the reaction was cooled at room temperature and was quenched with water (15 mL) and extracted with AcOEt. The organic layer was washed with a aqueous solution of HCl (10%) (2x10 ml) and brine (2x10 mL), then was concentrated under vacuum and the residue was purified by flash column chromatography on silica gel (5% AcOEt/hexane) to afford the two desired diastereosiomeric compounds (116 mg; 86/14; 99%). The title compound, was crystallized using a mixture of 30% AcOEt/hexane.

### Refinement

In (I) H atoms were placed in calculated positions and treated as ding atoms with C—H(tertiary), 0.98Å, C—H_2_(secondary), 0.97Å, C═ C—H_2_(terminal), 0.93Å, with *U*_iso_ = 1.2Ueq(C) and C—H(methyl), 0.96Å, with *U*_iso_ =1.5Ueq(C).

The H atoms attached to atom C11 were located on a final difference map. Atoms C8 and C9 show large thermal motion and, as a result, their contact distances to atom C7 are shorter than would be expected. Since the C7—C8 distance was longer than the C7—C9 distance C8 was assumed to be the methyl carbon. The H atoms attached to C8 and C9 could not be clearly seen on a final difference map.

Since no atom in the structure had an atomic number greater than 8 the absolute configuration could not be deterimed with Mokα radiation hence the Flack parameter is meaningless.

### Crystal data

**Table:**

C_11_H_15_NO	*D*_x_ = 1.061 Mg m^−^^3^
*M**_r_* = 177.24	Mo *K*α radiation, λ = 0.71073 Å
Orthorhombic, *P*2_1_2_1_2_1_	Cell parameters from 3155 reflections
*a* = 5.2892 (8) Å	θ = 2.8–26.9°
*b* = 10.7213 (16) Å	µ = 0.07 mm^−^^1^
*c* = 19.559 (3) Å	*T* = 293 K
*V* = 1109.1 (3) Å^3^	Prism, colourless
*Z* = 4	0.60 × 0.56 × 0.42 mm
*F*(000) = 384	

### Data collection

**Table:**

Bruker SMART CCD area-detector diffractometer	2590 independent reflections
Radiation source: fine-focus sealed tube	2112 reflections with *I* > 2σ(*I*)
Graphite monochromator	*R*_int_ = 0.023
phi and ω scans	θ_max_ = 27.9°, θ_min_ = 2.1°
Absorption correction: multi-scan (*SADABS*; Sheldrick, 1995)	*h* = −6→6
*T*_min_ = 0.861, *T*_max_ = 1.000	*k* = −13→13
7138 measured reflections	*l* = −19→25

### Refinement

**Table:**

Refinement on *F*^2^	Primary atom site location: structure-invariant direct methods
Least-squares matrix: full	Secondary atom site location: difference Fourier map
*R*[*F*^2^ > 2σ(*F*^2^)] = 0.052	Hydrogen site location: inferred from neighbouring sites
*wR*(*F*^2^) = 0.164	H-atom parameters constrained
*S* = 1.04	*w* = 1/[σ^2^(*F*_o_^2^) + (0.1051*P*)^2^ + 0.0575*P*] where *P* = (*F*_o_^2^ + 2*F*_c_^2^)/3
2590 reflections	(Δ/σ)_max_ < 0.001
120 parameters	Δρ_max_ = 0.24 e Å^−^^3^
0 restraints	Δρ_min_ = −0.20 e Å^−^^3^

### Special details

**Table:**

Experimental. ^1^H-NMR (CDCl_3_, δ):δ(eq) 4.83 (2*H*, J=26.07 Hz, H-9), 3.35–3.32 (1*H*, m, H-3), 2.82–2.74 (1*H*, m, H-5), 2.63–2.55 (2*H*, m, H-2 + H_eq_-4), 2.32–2.24 (2*H*, m, H_ax_-4 + H_eq_-6), 1.99–1.92 (1*H*, m, H_ax_-6), 1.77 (3*H*, d, J=6.70 Hz, H_3_-7) δ(ax) 4.94 (1*H*, s, H-9), 4.65 (1*H*, s, H-9), 2.84–2.81 (1*H*, m, H-5), 2.69–2.63 (2*H*, m, H-3 + H_eq_-4) 2.58- 2.49 (2*H*, m, H-2 + H_ax_-4), 2.35–2.18 (2*H*, m, H_eq + ax_-6), 1.75 (3*H*, s, H-10); 1.25 (3*H*, d, J=6.70 Hz, H-7). ^13^C-NMR (CDCl_3_, δ):δ(eq) 206.73 (C-1), 145.55 (C-8), 118.65 (C-11), 111.16 (CH_2_-9), 45.79 (CH_2_-4), 45.05 (CH-2), 42.25 (CH-5), 35.67 (CH-3), 32.85 (CH_2_-6), 20.53 (CH_3_-10), 12.59 (CH_3_-7); δ(ax) 207.23 (C-1), 144.76 (C-8), 120.54 (C-11), 113.88 (CH_2_-9), 46.70 (CH_2_-4), 40.35 (CH-5), 31.96 (CH-3), 30.52 (CH_2_-6), 21.89 (CH_3_-10), 13.50 (CH_3_-7). IR– (CDCl_3_, ν(cm^-1^)): 2974, 2936, 2359, 2237, 1709, 1447, 1379, 898. MS (EI^+^) (m/*z*, %): 162.99 ([C_11_H_14_O]^+^, 4); 178.12 ([*M*+1]^+^, 57); 200.10 ([*M*+ Na]^+^, 100); 201.05 (12); 201.10 (16); 210 (10); 216 (5). HRMS: 177.2429 calculated for C_11_H_15_ NO and found 177.1145.
Geometry. All e.s.d.'s (except the e.s.d. in the dihedral angle between two l.s. planes) are estimated using the full covariance matrix. The cell e.s.d.'s are taken into account individually in the estimation of e.s.d.'s in distances, angles and torsion angles; correlations between e.s.d.'s in cell parameters are only used when they are defined by crystal symmetry. An approximate (isotropic) treatment of cell e.s.d.'s is used for estimating e.s.d.'s involving l.s. planes.
Refinement. Refinement of *F*^2^ against ALL reflections. The weighted *R*-factor *wR* and goodness of fit *S* are based on *F*^2^, conventional *R*-factors *R* are based on *F*, with *F* set to zero for negative *F*^2^. The threshold expression of *F*^2^ > σ(*F*^2^) is used only for calculating *R*-factors(gt) *etc*. and is not relevant to the choice of reflections for refinement. *R*-factors based on *F*^2^ are statistically about twice as large as those based on *F*, and *R*- factors based on ALL data will be even larger.

### Fractional atomic coordinates and isotropic or equivalent isotropic displacement parameters (Å^2^)

**Table:**

	*x*	*y*	*z*	*U*_iso_*/*U*_eq_	
O	0.1359 (3)	0.16426 (13)	0.97088 (8)	0.0752 (4)	
N	0.1851 (4)	−0.20500 (17)	0.92433 (12)	0.0888 (6)	
C1	0.5822 (3)	−0.06508 (16)	0.94292 (9)	0.0573 (4)	
H1	0.7132	−0.1143	0.9659	0.069*	
C2	0.5121 (4)	0.04653 (16)	0.99035 (8)	0.0588 (4)	
H2	0.6689	0.0917	1.0002	0.071*	
C3	0.3426 (3)	0.13420 (15)	0.95095 (9)	0.0564 (4)	
C4	0.4468 (4)	0.17830 (18)	0.88397 (11)	0.0705 (5)	
H4A	0.5991	0.2266	0.8919	0.085*	
H4B	0.3243	0.2320	0.8617	0.085*	
C5	0.5082 (4)	0.06743 (17)	0.83708 (9)	0.0625 (4)	
H5	0.3501	0.0222	0.8288	0.075*	
C6	0.6881 (4)	−0.02143 (19)	0.87410 (10)	0.0646 (5)	
H6A	0.8484	0.0203	0.8816	0.078*	
H6B	0.7192	−0.0935	0.8454	0.078*	
C7	0.6110 (5)	0.1083 (2)	0.76790 (12)	0.0885 (7)	
C8	0.7980 (8)	0.2010 (4)	0.76281 (18)	0.1574 (17)	
H8A	0.7514	0.2597	0.7280	0.236*	
H8B	0.9568	0.1631	0.7511	0.236*	
H8C	0.8137	0.2434	0.8058	0.236*	
C9	0.5180 (13)	0.0550 (7)	0.71081 (16)	0.263 (4)	
H9A	0.5776	0.0796	0.6681	0.316*	
H9B	0.3943	−0.0063	0.7141	0.316*	
C10	0.3602 (4)	−0.14556 (16)	0.93301 (10)	0.0642 (5)	
C11	0.4019 (6)	0.0058 (2)	1.05820 (10)	0.0901 (7)	
H11A	0.2379	−0.0301	1.0509	0.135*	
H11B	0.3870	0.0768	1.0879	0.135*	
H11C	0.5111	−0.0550	1.0789	0.135*	

### Atomic displacement parameters (Å^2^)

**Table:**

	*U*^11^	*U*^22^	*U*^33^	*U*^12^	*U*^13^	*U*^23^
O	0.0629 (8)	0.0698 (8)	0.0928 (10)	0.0087 (7)	0.0027 (7)	−0.0158 (7)
N	0.1012 (14)	0.0644 (10)	0.1009 (14)	−0.0174 (11)	0.0103 (12)	−0.0007 (9)
C1	0.0587 (9)	0.0538 (8)	0.0594 (9)	0.0115 (7)	0.0018 (7)	−0.0007 (7)
C2	0.0646 (10)	0.0575 (8)	0.0542 (8)	0.0038 (8)	−0.0016 (8)	−0.0046 (7)
C3	0.0598 (9)	0.0453 (7)	0.0641 (9)	−0.0001 (7)	−0.0026 (8)	−0.0111 (6)
C4	0.0820 (13)	0.0565 (9)	0.0729 (11)	0.0033 (9)	−0.0021 (10)	0.0073 (8)
C5	0.0656 (10)	0.0669 (10)	0.0549 (9)	−0.0066 (9)	0.0005 (8)	0.0009 (8)
C6	0.0609 (10)	0.0682 (10)	0.0647 (9)	0.0022 (9)	0.0111 (8)	−0.0039 (8)
C7	0.1031 (17)	0.0994 (15)	0.0630 (11)	−0.0087 (15)	0.0083 (12)	0.0114 (11)
C8	0.179 (3)	0.205 (4)	0.0891 (19)	−0.075 (4)	0.014 (2)	0.044 (2)
C9	0.358 (9)	0.365 (8)	0.0663 (17)	−0.233 (8)	0.047 (3)	−0.045 (3)
C10	0.0789 (12)	0.0467 (8)	0.0669 (10)	0.0016 (9)	0.0094 (9)	0.0003 (7)
C11	0.129 (2)	0.0840 (13)	0.0578 (10)	0.0202 (15)	0.0180 (12)	−0.0017 (10)

### Geometric parameters (Å, º)

**Table:**

O—C3	1.205 (2)	C5—C6	1.529 (3)
N—C10	1.137 (3)	C5—H5	0.9800
C1—C10	1.470 (3)	C6—H6A	0.9700
C1—C6	1.531 (3)	C6—H6B	0.9700
C1—C2	1.559 (2)	C7—C9	1.347 (5)
C1—H1	0.9800	C7—C8	1.406 (5)
C2—C3	1.510 (2)	C8—H8A	0.9600
C2—C11	1.513 (3)	C8—H8B	0.9600
C2—H2	0.9800	C8—H8C	0.9600
C3—C4	1.498 (3)	C9—H9A	0.9300
C4—C5	1.536 (3)	C9—H9B	0.9300
C4—H4A	0.9700	C11—H11A	0.9600
C4—H4B	0.9700	C11—H11B	0.9600
C5—C7	1.522 (3)	C11—H11C	0.9600
			
C10—C1—C6	110.83 (16)	C4—C5—H5	107.5
C10—C1—C2	109.82 (15)	C5—C6—C1	112.29 (15)
C6—C1—C2	112.06 (14)	C5—C6—H6A	109.1
C10—C1—H1	108.0	C1—C6—H6A	109.1
C6—C1—H1	108.0	C5—C6—H6B	109.1
C2—C1—H1	108.0	C1—C6—H6B	109.1
C3—C2—C11	113.48 (17)	H6A—C6—H6B	107.9
C3—C2—C1	108.38 (13)	C9—C7—C8	119.8 (3)
C11—C2—C1	113.08 (16)	C9—C7—C5	119.0 (3)
C3—C2—H2	107.2	C8—C7—C5	121.2 (2)
C11—C2—H2	107.2	C7—C8—H8A	109.5
C1—C2—H2	107.2	C7—C8—H8B	109.5
O—C3—C4	122.18 (18)	H8A—C8—H8B	109.5
O—C3—C2	122.69 (18)	C7—C8—H8C	109.5
C4—C3—C2	115.10 (16)	H8A—C8—H8C	109.5
C3—C4—C5	110.84 (15)	H8B—C8—H8C	109.5
C3—C4—H4A	109.5	C7—C9—H9A	120.0
C5—C4—H4A	109.5	C7—C9—H9B	120.0
C3—C4—H4B	109.5	H9A—C9—H9B	120.0
C5—C4—H4B	109.5	N—C10—C1	178.0 (2)
H4A—C4—H4B	108.1	C2—C11—H11A	109.5
C7—C5—C6	112.20 (17)	C2—C11—H11B	109.5
C7—C5—C4	112.55 (17)	H11A—C11—H11B	109.5
C6—C5—C4	109.32 (15)	C2—C11—H11C	109.5
C7—C5—H5	107.5	H11A—C11—H11C	109.5
C6—C5—H5	107.5	H11B—C11—H11C	109.5
			
C10—C1—C2—C3	−71.53 (18)	C3—C4—C5—C6	−55.5 (2)
C6—C1—C2—C3	52.1 (2)	C7—C5—C6—C1	−178.64 (18)
C10—C1—C2—C11	55.2 (2)	C4—C5—C6—C1	55.8 (2)
C6—C1—C2—C11	178.83 (19)	C10—C1—C6—C5	67.67 (19)
C11—C2—C3—O	−2.8 (2)	C2—C1—C6—C5	−55.4 (2)
C1—C2—C3—O	123.73 (18)	C6—C5—C7—C9	102.3 (5)
C11—C2—C3—C4	179.06 (17)	C4—C5—C7—C9	−133.9 (5)
C1—C2—C3—C4	−54.45 (19)	C6—C5—C7—C8	−78.4 (3)
O—C3—C4—C5	−120.6 (2)	C4—C5—C7—C8	45.4 (4)
C2—C3—C4—C5	57.6 (2)	C6—C1—C10—N	−58 (6)
C3—C4—C5—C7	179.12 (18)	C2—C1—C10—N	66 (6)
